# Combination therapy – a way to forestall artemisinin resistance and optimize uncomplicated malaria treatment

**Published:** 2015

**Authors:** I Ștefan

**Affiliations:** * Infectious Diseases Ward, ”Dr. Carol Davila” Central Military University Emergency Hospital, Bucharest, Romania

**Keywords:** Artemisinin resistance, ACT, Plasmoquine, Pyronaridine, kelch 13

## Abstract

Artemisinin resistance represents a global concern, which requires a concerted and coordinated effort at a global level. Lessons learned from the experience of drug combination therapies in HIV, TB, and HCV infections showed that combination therapies reduce the risk of drug resistance development. In order to maximize the effectiveness of artemisinin and its derivates and to protect it from the development of resistance, WHO recommended that they should be combined with other drugs that have different mechanisms of action and longer half-lives. Until the attainment of new pharmaceuticals, artemisinin-based combination therapy (ACT) is the way to forestall artemisinin resistance and optimize uncomplicated malaria treatment.

Plasmodium resistance to antimalarial drugs is one of the major obstacles in the fight against malaria. Of all the countries in which Plasmodium falciparum malaria exists, only those in Central America (Honduras, Nicaragua, Haiti, The Dominican Republic) and most of the Middle East (except for Iran, Oman, Saudi Arabia, Yemen) have not recorded resistance to chloroquine. The rapid spread and development of chloroquine resistance throughout the world was followed by the development of resistance to sulfadoxine/ pyrimethamine (Fansidar®) in Southeast Asia, South America and, locally, in Africa. It was proved that, in Thailand, more than 50 percent of the cases in certain border areas no longer respond to mefloquine, while the sensitivity to quinine is also diminishing in areas of Thailand and Vietnam.

Resistant strains of P. vivax have been reported in Papua New Guinea, Indonesia, Thailand, India, Africa (most of the strains) and few cases have recently been observed in Vanuatu. High resistance to Maloprim and Halofantrine has also been reported. Recently, a P. falciparum isolate from Nigeria has been found resistant to Malarone®. In Africa, there is evidence that the spread of resistance coincided with increases in child mortality and morbidity [**1**]. However, the emergence of artemisinin resistance in the Greater Mekong subregion (GMS) [**2**] is the matter of paramount concern. That is because there is no alternative antimalarial treatment currently available to offer the same level of efficacy and tolerability as artemisinin-based combination therapies (ACTs). 

The treatment of uncomplicated malaria consists of oral therapy with a combination of at least two agents. The purpose of this strategy is to forestall the development of further antimalarial resistance and protect the agents that are presently effective. Local antimalarial drug resistance, tolerability, availability, and gametocidal activity guide the selection of the appropriate therapeutic agents.

In order to maximize the effectiveness of artemisinin and its derivates and to protect it from the development of resistance, WHO repeatedly recommended that they should be combined with other drugs that have different mechanisms of action and longer half-lives (WHO 2001, 2006, 2010). ACTs are recommended by the WHO as the first-line treatment of uncomplicated falciparum malaria [**3**]. There are currently five ACTs recommended by WHO [**4**]: artemether + lumefantrine, artesunate + amodiaquine, artesunate + mefloquine, artesunate + sulfadoxine-pyrimethamine, and dihydroartemisinin + piperaquine. All ACTs contain an artemisinin derivate combined with a partner drug. The role of the artemisinin compound is to reduce the parasite load rapidly during the first days of treatment while the role of the partner drug is to eliminate any remaining parasites. In addition, the probability of emergence of a spontaneous mutation that confers resistance to both drugs (having unrelated mechanisms of action) is very low [**5**]. Artemisinins should be given in combination to prevent resistance and the second agent must have a longer half-life to provide an extended duration of drug level in order to cure the infection [**6**,**7**]. However, P. falciparum resistance has been detected in four countries in GMS: Cambodia, Myanmar, Thailand, and Vietnam. As chloroquine resistance divided the malarial map in three zones, artemisinin claimed the classification of geographical areas into tiers of artemisinin resistance:

**Tier I:** Areas where there is credible evidence of artemisinin resistance.

**Tier II:** Areas with significant inflows of people from tier I areas, including those immediately bordering tier I.

**Tier III:** Areas with no evidence of artemisinin resistance and limited contact with tier I areas [**8**].

Recently, a molecular marker of artemisinin resistance was identified. Mutations in the parasite’s kelch protein gene on chromosome 13 (kelch 13) were shown to be associated with delayed parasite clearance in vitro and in vivo [**9**] and with P. falciparum artemisinin resistance across Southeast Asia. In the GMS, the most prevalent mutation was the C580Ymutation, but many others in and near K13 propeller region were found to be associated with artemisinin resistance (Y493H, R539T, I543T). The prevalence of artemisinin K13 propeller varies greatly by region rates as high as 47 percent in parts of Myanmar. 

Spread of artemisinin resistant parasites is due to transmission of single clones across region; however, individual populations of parasites can develop artemisinin resistance independently, which may make containment of artemisinin resistance more challenging [**10**,**11**].

Based on the latest data resulted from therapeutic efficacy studies of ACTs, clinical trials using artesunate monotherapy as well as K13 sequencing, in August 2014, WHO established the new definition of artemisinin resistance:

**Suspected** partial artemisinin resistance is defined as: 

• ≥ 5% of patients carrying K13 resistance-associated mutations; 

or 

• ≥ 10% of patients with persistent parasitemia by microscopy on day 3 after treatment with ACT or artesunate monotherapy; 

or 

• ≥ 10% of patients with a parasite clearance half-life of ≥ 5 hours after treatment with ACT or artesunate monotherapy. 

**Confirmed** partial artemisinin resistance is defined as:

• ≥ 5% of patients carrying K13 resistance-associated mutations, all of whom have been found, after treatment with ACT or artesunate monotherapy, to have either persistent parasitemia by microscopy on day 3, or a parasite clearance half-life of ≥ 5 hours.

The term “partial artemisinin resistance” is used to describe delayed parasite clearance observed while following treatment with artesunate monotherapy or after treatment with an ACT regimen. Delayed parasite clearance will not necessarily lead to treatment failure. In the GMS, high treatment failure rate following treatment with an ACT has only been observed where resistance to the partner drug existed, regardless of the presence of artemisinin resistance. In Thailand and Cambodia, failure to artemisinin-mefloquine therapy was reported only in areas with high prevalence of mefloquine resistance. Also, in Cambodia, treatment with dihydroartemisinin-piperaquine failed because of the emergence of piperaquine resistance. WHO recommends monitoring the efficacy of first-line and second-line ACTs at every two years in all malaria falciparum endemic countries. The reports must specify the proportion of patients who are parasitemic on day 3 and the proportion of treatment failure by 28 or 42 day (depending on the used ACT) [**12**].

In the interest of containing and eliminating artemisinin resistance, in 2011, WHO elaborated the Global plan for artemisinin resistance containment (GPARC). The GPARC was developed in response to the identification of artemisinin-resistance in the border region between Cambodia and Thailand and the concern that it could spread and/ or emerge spontaneously elsewhere. The primary objective of GPARC is to protect ACTs as an effective treatment for P. falciparum malaria [**13**]. 

Artemisinin resistance represents a global concern, which requires a concerted and coordinated effort at a global level. Lessons learned from the experience of drug combination therapies in HIV, TB, and HCV infections showed that combination therapies reduce the risk of drug resistance development. 

Patients who received monotherapy with rapidly eliminating agents are exposed to an increased risk of relapse. Those treated with a low-acting agent in monotherapy have slower parasite or fever clearance and longer symptom duration. 

In areas threatened by artemisinin resistance, a single gametocidal dose of primaquine added to the ACT regimen is recommended as an adjunct for the reduction of malaria transmission [**14**,**15**-**18**]. The addition of a gametocidal drug such as primaquine to an ACT may provide a supplementary benefit. Artemisinin derivates have activity against younger gametocytes but appear inactive against mature gametocytes against which primaquine is very effective. The addition of a single dose of primaquine (0.75 mg/ kg) to ACT has substantially reduced P. falciparum gametocyte carriage and was also well tolerated [**19**]. Likewise, it has been proved that the two medicines act synergistically [**20**].

In 2012, the WHO recommended the administration of a reduced dose of primaquine (0.25 mg base/ day) on the first day of treatment with ACT in adults (except for pregnant women) and children >12 months, in areas threatened by artemisinin resistance, in order to prevent toxicity in patients with G6PD deficiency. In areas where clinicians already use a single primaquine dose of 0.75 mg base/ kg, this practice should be continued until further information is available [**15**]. The recommendation also stated that primaquine dosing of 0.25 mg base/ kg is unlikely to cause serious toxicity in patients with G6PD deficiency so may be administered in the absence of enzyme testing.

Recently, a new artemisinin-based combination therapy regimen has been developed – pyronaridine-artesunate. Pyronaridine is a mannich base that was synthesized in China in 1970 and has been widely used alone or in combination for the treatment of uncomplicated malaria in the same country. A randomized trial including 1272 children and adults from Africa and Asia demonstrated that pyronaridine-artesunate regimen resulted in a 28-day complete response rate of 99 percent [**21**]. Pyronaridine-artesunate was prescribed as single daily dose taken for three days; patients on this regimen had a significantly lower rate of new infections on day 28 and day 42, likely due to the prolonged half-life of the pyronaridine component. The single day dosing and greater efficacy makes pyronaridine-artesunate an excellent addition to the ACT armamentarium [**21**].

In conclusion, the priorities in the coming years are:

• Increased monitoring of antimalarial drug efficacy and resistance

• Use of molecular markers to identify genetic mutations related to antimalarial drug resistance in the parasite genome

• Use of gametocidal drugs for the reduction of malaria transmission 

• Attainment of additional pharmaceuticals 

• Use of combination therapy to forestall drug resistance.
